# Mapping Vaccine Sentiment by Analyzing Spanish-Language Social Media Posts and Survey-Based Public Opinion: Dual Methods Study

**DOI:** 10.2196/63223

**Published:** 2025-08-29

**Authors:** Agnes Huguet-Feixa, Wasim Ahmed, Eva Artigues-Barberà, Joaquim Sol, Xavier Gomez-Arbones, Pere Godoy, Marta Ortega Bravo

**Affiliations:** 1 Centre d'Atenció Primària Rambla Ferran, Institut Català de la Salut, Lleida, Spain; 2 Grup de Recerca multidisciplinari en Terapèutica i Intervencions en Atenció Primària (Grup RETICAP), Lleida, Spain; 3 Faculty of Medicine, University of Lleida, Lleida, Spain; 4 Fundació Institut Universitari per a la recerca a l'Atenció Primària de Salut Jordi Gol i Gurina (IDIA-PJGol), Gran Via de les Corts Catalanes, 587, Barcelona, 08007, Spain; 5 Hull University Business School, Hull, United Kingdom; 6 Atenció Primària, Gerència Territorial de Lleida, Institut Català de la Salut, Lleida, Spain; 7 Facultat d’Infermeria i Fisioteràpia, Universitat de Lleida, Lleida, Spain; 8 Department of Experimental Medicine, Institut de Recerca Biomèdica de Lleida, University of Lleida, Lleida, Spain; 9 Departament de Salut, Hospital Universitario Santa Maria, Gestió de Serveis Sanitaris, Lleida, Spain; 10 Centro de Investigación Biomédica en Red de Epidemiologia y Salud Publica, Instituto de Salud Carlos III, Madrid, Spain; 11 Institut de Recerca Biomèdica de Lleida, Applied Epidemiology Research Group, University of Lleida, Lleida, Spain; 12 Grup de Recerca en Ecografia Clínica d’Atenció Primària (Grup GRECOCAP), Lleida, Spain; 13 Centre d'Atenció Primària Almacelles, Atenció Primària, Gerència Territorial de Lleida, Institut Català de la Salut, Carrer Melcior de Guàrdia, 36, Almacelles, Lleida, 25100, Spain, 34 973 74 20 20

**Keywords:** anti-vaccination movement, vaccination refusal, social media, negative sentiment, survey, misinformation, internet, immunization, vaccine hesitancy, communication, public opinions, health information, Spain, Spanish

## Abstract

**Background:**

The internet and social media have been considered useful platforms for obtaining health information. However, critical and erroneous content about vaccines on social media has been associated with vaccination delays and refusal.

**Objective:**

This study aimed to examine how social networks influence access to and perceptions of vaccine-related information. We sought to (1) quantify the proportion of individuals engaging with vaccine-related content on social media and to characterize their demographic and behavioral profiles through an internet-based population survey conducted in Spain and (2) to analyze vaccine-related sentiments and opinions in Spanish and Catalan posts on X (X Corp [formerly Twitter, Inc] and geolocate them using artificial intelligence.

**Methods:**

Two complementary methodologies were applied. First, an observational study was conducted via a self-administered internet-based questionnaire among adults in Spain in 2021. Second, we analyzed Spanish- and Catalan-language posts from X, collected between March and December 2021. Sentiment analysis was performed using a workflow developed in Orange Data Mining (Bioinformatics Laboratory, Faculty of Computer and Information Science, University of Ljubljana). Geolocation was based on user-defined locations and visualized using Microsoft Power Business Intelligence. Social network analysis was conducted with NodeXL Pro (Social Media Research Foundation) to identify and characterize the 5 largest user communities discussing vaccines. Although based on independent data sources, the 2 approaches provided complementary methodological insights.

**Results:**

Among the 1312 respondents in the survey, 85.7% (1124/1312) stated that they were regular social network users, and 66% (850/1287) reported having encountered antivaccine information on social networks. Of these, 24.3% (205/845) experienced doubts about receiving recommended vaccines, and out of those with doubts, 13.3% (27/203) refused at least 1 vaccine proposed by a health care professional. A total of 479,734 Spanish and Catalan posts on X were analyzed, with 54.44% (n=261,183) posts classified as negative, 28.18% (n=135,194) as neutral, and 17.37% (n=83,357) as positive. Sentiment varied across regions, with more negative posts appearing to derive from South America, with a mix in Europe and more positive posts in North America. Analysis of the topic words and key themes allowed the grouping of the predominant themes of the 5 study groups, which were (1) vaccination efforts during the COVID-19 pandemic, (2) issues of vaccine theft and struggles in managing and securing the vaccine supply, (3) campaigns in the State of Mexico, (4) vaccination efforts for older adults, and (5) the vaccination campaign in Colombia to combat COVID-19.

**Conclusions:**

High proportions of exposure to antivaccine content were reported by the surveyed population. Sentiment analysis and geolocation of posts on the social network X suggested a notable presence of Spanish-language posts categorized as negative, predominantly from South America. The thematic analysis of conversations on X may provide valuable insights into the population’s opinions about vaccines.

## Introduction

The critical and erroneous content about vaccines on social networks has posed a significant obstacle to immunizing the population against vaccine-preventable diseases [1-7]. A decline in vaccination coverage represents a threat to the herd immunity acquired in recent years through the efforts of health care professionals and could lead to outbreaks of diseases, such as the measles outbreak in Europe in 2023‐2024 [8].

The crisis in the vaccination system and the resurgence of antivaccine movements are due to the increased accessibility of misinformation and the reduced credibility of health care personnel [9-11]. The internet and social media have changed the way the public accesses health information [12,13]. This information on social networks frequently contains incorrect data about vaccine effectiveness or data from antivaccine movements, which can influence the decision to reject vaccination before individuals even set foot in primary care centers [13,14]. A study published in 2020 stated that Facebook (Meta) pages that distrust established health guidelines are more effective at influencing hesitant individuals than government agencies [15]. Distrust in scientific positions could spread and dominate social network conversations over the next decade [15]. Exposure to antivaccine content on social media has been associated with delays and rejection of vaccination and is considered one of the major causes of vaccine hesitancy [3,16-19].

Current literature has yet to explain how the antivaccine movement continues to engage and persuade the public to reject immunization despite the efforts of vaccine advocates [16]. Antivaccine proponents on social networks have shown more active engagement patterns than provaccine groups. In a study published in 2020, analyzing Facebook users, it was shown that antivaccine groups on social networks, though a minority, had the potential to be more influential than provaccine groups. Moreover, undecided groups were not passive; they were the most active agents in the discussion. This fact favored the interaction of the undecided with antivaccine groups [15], and for this reason, intervention strategies were proposed to identify central and influential antivaccine groups to reduce their growth and the formation of future antivaccine or undecided groups [15].

X (formerly known as Twitter) has been an important source of information for studying vaccine hesitancy, as social media platforms have been considered effective tools for communication between individuals and organizations, but they have also been used as tools to spread false information and conspiracy theories about vaccines [20-22].

Several studies have been published analyzing public opinions or sentiments about vaccines on X using artificial intelligence (AI). These publications suggested that the analysis of social networks using AI should be considered by institutions and governments alongside surveys and other conventional methods to assess public attitudes toward vaccines [16,21,23-25]. AI techniques, such as natural language processing and machine learning, enable real-time sentiment analysis of social media posts, identifying shifts in public opinion linked to events or misinformation [21,24,26]. Social bots on platforms like Twitter can shape vaccine perceptions by amplifying both positive and negative sentiments [27,28]. AI tools track sentiment trends over time and across regions, offering insights into geographical variations in public opinion [21,24,25]. These insights can support real-time assessments of public confidence in vaccines, address the concerns of skeptics, and inform more effective communication strategies to enhance vaccine acceptance [24,29].

A prior study combining survey data with social media analysis, conducted in the context of influenza vaccination in the United States, illustrates a precedent for using these 2 methodologies in parallel. This study analyzed geolocated tweets using topic modeling techniques alongside individual-level survey data on vaccination attitudes and behaviors, offering insights into potential associations between social media content and offline public health outcomes [30]. In this line, our study applies a dual-methods design, seeking to capture complementary perspectives on public discourse and sentiment.

Our study aimed to determine how social networks influence access to and perception of vaccine-related information. To this end, we established the following objectives: (1) to quantify the proportion of individuals engaging with vaccine-related content on social media and to characterize their demographic and behavioral profiles through an internet-based population survey conducted in Spain and (2) to analyze vaccine-related sentiments and opinions in Spanish and Catalan posts on X, and geolocate them using AI.

## Methods

### Overview

This dual-methods design was developed to obtain a comprehensive exploration of vaccine-related communication by analyzing 2 independent but complementary data sources. The quantitative component consisted of a survey administered to adults residing in Spain, capturing self-reported attitudes and behaviors regarding vaccines. In parallel, the second component involved the collection and analysis of Spanish- and Catalan-language posts published globally on X. Both data strands were analyzed separately, involving distinct populations, and no direct linkage between participants was established. While the survey yielded structured quantitative data on personal vaccine-related behaviors and attitudes, the social media analysis provided insight into naturally occurring, public vaccine narratives and expressions within social mediacommunities. The parallel, though independent, implementation of these methods allowed for an exploratory examination of how vaccine-related narratives are both consumed via self-report and expressed through spontaneous discourse, thereby offering different but complementary perspectives on the phenomenon without implying a formal methodological integration.

### Survey Data Collection and Analysis

#### Study Design

We conducted an observational, cross-sectional study on individuals who had access to an internet-based survey from March to December 2021 (Multimedia Appendix 1). The inclusion criteria required participants to be aged 18 years or older and have the authority to make vaccination decisions for themselves or others in Spain. We obtained the information through an electronic, self-administered questionnaire designed by the project research team. A pilot test was performed before the definitive questionnaire was obtained. To ensure the rigor and validity of the study, an experienced research team with expertise in conducting surveys will be needed, thereby guaranteeing the quality and reliability of the instrument. Both were registered on a REDCap (Research Electronic Data Capture) web platform on a centralized server where the data remain in the custody of the Institut Català de la Salut (Catalan Health Institute). Through the REDCap web platform, we also built a database of the participants. Anonymous information was exported to the statistical packages used for subsequent analysis. The REDCap platform generated a link [31] for participation in the survey that was disseminated through scientific societies, social media, research institutes, pediatricians, and nurses in primary care.

#### Variables

The main variables of the questionnaire are listed in Textbox 1.

Textbox 1. **Variables of the questionnaire.**

**Sociodemographic factors**

- Sex
- Age
- Having children younger than 15 years
- Level of education

**Information on the use of social network**

- Use of social network and duration of use.
- Whether information about vaccines was sought on social networks.
- Which social network was used to search for vaccine information?
- The year of the first search for vaccines on social networks.
- Whether the search was related to COVID-19.

**Antivaccine information on social network**

- Whether they had encountered the antivaccine information.
- Whether such information had caused doubts.
- The type of doubts.
- Whether the information on social networks had led them to reject vaccines.
- Whether vaccines were rejected for a child and, if so, the child’s age.
- Who initiated the doubt, and the gender, age, and education level of the person rejecting vaccination.

**Opinions about vaccines on social network**

- Whether they had received opinions against vaccines.
- Whether they had received opinions in favor of vaccines.
- Whether they followed a social network that generated doubts about vaccines or profiles against or in favor of vaccination.
- Whether they had made any comments against or in favor of vaccines on social networks.

#### Statistical Analysis

The data were gathered in an anonymized database using the REDCap platform. We conducted a descriptive analysis of the findings, categorizing qualitative or ordinal data using absolute and relative frequencies. The analysis was complemented within selected subgroups of interest to identify the factors independently associated with these results. Statistical analyses were performed with R software version 4.1.2 (R Foundation for Statistical Computing). In the statistical analysis, only complete cases were considered, and missing values were assumed to be missing at random.

### X Insights and Social Network Analysis

#### Data Retrieval

Posts were gathered from X. The search string in order to retrieve posts was as follows: “vacunacion OR vacunacio OR vacunas OR vacunes OR antivacunas OR antivacunes OR antivacinacion OR antivacunacio.” We relied on Spanish and Catalan keywords to retrieve our dataset, and geolocation retrieval was not used. Our data retrieval would also include hashtag versions of these keywords as well as any replies to posts that would have used these keywords. A total of 479,734 posts were captured (excluding reposts), and these were sent by 29,706 users. The step of excluding reposts greatly enhanced the clarity of the dataset. Due to the difficulties in assessing spam or “bots,” no further processing was applied. The data were randomly sampled between March 2021 and December 2021 (see Table 1). This time period is selected because it aligns with the second phase of the COVID-19 vaccine, when vaccines were becoming more accessible to the general public. This 10-month window offers an overview during this important period.

**Table 1.** Overview of data (tweet volumes and total number of X users).

Month (2021)	Total number of posts	Total number of X users
March	54,837	40,093
April	53,793	39,426
May	53,613	39,114
June	54,499	38,590
July	54,074	40,170
August	54,002	39,303
September	47,141	33,402
October	35,071	25,545
November	35,417	27,944
December	37,287	29,706
Total	479,734	—^a^

a Not applicable

#### Sentiment Analysis

Orange Data Mining was used to create a workflow for sentiment analysis of the 479,734 posts that were captured. Sentiment analysis aims to calculate the sentiment for each post within the dataset. The study drew upon the multilingual sentiment lexicon built into Orange Data Mining. This sentiment analysis is based on unsupervised learning. Orange Data Mining draws upon a multilingual lexicon derived from affective norms for words across several languages, including Spanish. Lexicons are essentially lists of positive and negative words that can be used to determine the overall sentiment of a text. These are embedded in Orange Data Mining, and they can be downloaded elsewhere [32]

Moreover, in Orange Data Mining, depending on the content of each post, a score is provided to each post, such as −10 (ie, negative), 6 (which would be positive), and 0 (neutral). These scores are based on the positive and negative lexicons within the posts. An average sentiment score was also calculated by computing the average sentiment score of all 479,734 posts.

Although this sentiment analysis relies on a general-purpose lexicon, we acknowledge that in the context of vaccine-related communication, negative sentiment may encompass a broad spectrum of emotional expressions. These include not only rejection or hostility but also more nuanced affective states, such as concern, fear, uncertainty, grief, or anger. This is particularly relevant in vaccine discourse, where emotional responses may reflect hesitancy, confusion, or distress rather than outright opposition.

#### Location-Based Analysis

Location-based analysis was conducted by drawing upon self-defined user locations from X, entered into Microsoft Power Business Intelligence for analysis. A challenge with self-defined locations (ie, user-reported) is that as they allow users to enter free text, they may provide a city or region rather than the country. These are, therefore, better analyzed visually. Data were collected from April to December 2021. Location analysis focused on examining a specific time period in our dataset, that is, April 2021.

#### Social Network Analysis and Topic Insight

Social network analysis was conducted to identify the nature of the top 5 groups conversing about vaccinations in Spanish, drawing upon NodeXL Pro. Social network and topic analysis focused on examining April 2021, which provided a snapshot of key influencers at the start of our time. April was specifically selected because this was when the pace of vaccinations began to increase and mass vaccination centers began to upscale their operations.

Our study used NodeXL Pro to conduct social network analysis, examining how different users interacted with each other to identify patterns in interactivity. This included how users replied, retweeted, and mentioned each other. When these interaction patterns are analyzed in aggregate, they uncover hidden insights. These patterns were then used to assign each of the users to distinct “groups.” By analyzing the position of users within the network using betweenness centrality, it allowed the ability to identify influential users within the network. Our approach followed that of previous research [33]. In terms of specific methodological steps, the networks were laid out using the “Group in the Box” format. The graphs were directed. The graph’s vertices were grouped by cluster using the Clauset-Newman-Moore cluster algorithm [34]. The graph was laid out using the Harel-Koren Fast Multiscale layout algorithm [35]. By examining the most popular words, key posts, and tweets within the dataset, we assigned various themes to each of the groups. Using the above insights, we were able to identify the top 5 users within the network, drawing upon betweenness centrality to the key influencers within the network. An additional insight gained is the type of words being used by users within the network. Our analysis provides insight into the 2 words most often used together. This helps shed light on the issues users may have been talking about.

### Ethical Considerations

This study was approved by the ethics and clinical Research Committee of the Institut Universitari per a la recerca a l’Atenció Primària de Salut (University Institute for Research in Primary Health Care; IDIAP) Jordi Gol i Gurina, with code 20/221-P. The study was conducted in accordance with the principles of the Declaration of Helsinki. The variables collected were treated anonymously to guarantee the confidentiality of the data, as established in Regulation (European Union) 2016/679 of the European Parliament and the Council of April 27 on Data Protection (General Data Protection Regulation) and the organic law 3/2018, of December 5, on the protection of personal data and the guarantee of digital rights. The database is kept by the principal investigator and the research team in an Excel (Microsoft) format, protected by password access. An anonymized database was used for the analysis. Before carrying out the survey, internet-based informed consent had to be completed, accepted, and signed. Participation was voluntary, and no compensation was provided to participants.

## Results

### Survey Outcomes

A total of 1312 respondents were analyzed, with 74.5% (954/1280) being female, 71.0% (915/1289) university graduates, 46.9% (604/1289) having children younger than 15 years of age, 14.0% (180/1287) aged 30 years or younger, 12.4% (159/1287) older than 60 years of age, and 73.7% (948/1287) between 31 and 59 years old. Among the respondents, 85.7% (1124/1312) stated they were regular social network users and shared sociodemographic characteristics (age, children, gender, and education) similar to the total sample analyzed (Table 2), with a predominance of females and university education. Of the regular social network users, 76% (852/1121) had been using social network for more than 5 years, and 35.6% (399/1121) reported seeking information about vaccines on social network. Among these, 39.1% (156/399) used X, 29.6% (118/399) used Instagram, 33.1% (132/399) used Facebook, and 46.6% (186/399) used other social networks. A total of 53.1% (196/369) reported conducting their first vaccine search on social network in 2020, with 84.1% (329/391) stating that their search was related to the COVID-19 pandemic.

**Table 2.** Participant characteristics and their engagement with social media regarding vaccine information.

Survey questions	Value (N*=*1312)	Responders, n
Sex, n (%)		1280
Male	326 (25.5)	
Female	954 (74.5)	
Age (year), n (%)		1287
30 years or less	180 (14.0)	
31-59 years	948 (73.7)	
60 years or more	159 (12.4)	
University studies, n (%)	915 (71.0)	1289
Do you have children aged 14 or younger?, n (%)	604 (46.9)	1289
Do you regularly use social networks?, n (%)	1124 (85.7)	1312
Since when?, n (%)		1121
Less than 1 year ago	13 (1.16)	
Since Covid-19 lockdown	18 (1.61)	
1-5 years	238 (21.2)	
More than 5 years	852 (76.0)	
Have you searched for vaccine information on social networks?, n (%)	399 (35.6)	1121
On which social networks?, n (%)		399
Twitter/X	156 (39.1)	
Instagram	118 (29.6)	
Facebook	132 (33.1)	
Others	186 (46.6)	
In which year did you first search for vaccine information on social networks?, n (%)		369
2020	196 (53.1)	
Before 2020	154 (41.7)	
After 2020	19 (5.15)	
Was it related to COVID-19?	329 (84.1)	391
Have you found information AGAINST vaccines on social networks?, n (%)	850 (66.0)	1287
Did it generate any doubts about the recommended vaccination?, n (%)	205 (24.3)	845
Has information from social networks led you to reject any vaccine proposed by a healthcare professional?, n (%)	27 (13.3)	203
If you have rejected vaccinating a child, what were their ages?, mean (SD)	9.11 (7.41)	9
If you have rejected vaccinating a child, who initially had the doubt?, n (%)		15
Myself	13 (86.7)	
Another person	2 (13.3)	
Sex		17
Male	5 (29.4)	
Female	10 (58.8)	
Other	2 (11.8)	
Age (year)		22
Less than 30	5 (22.7)	
30-39	2 (9.09)	
40-49	6 (27.3)	
50-59	7 (31.8)	
60-69	1 (4.55)	
More than 70	1 (4.55)	
Highest educational level completed by the person who rejected vaccination		25
No education or incomplete primary studies	1 (4.00)	
Secondary	3 (12.0)	
High School	3 (12.0)	
Vocational training programs	1 (4.00)	
University	17 (68.0)	
Have you received any type of opinion or comment through social networks? n (%)		1287
Against vaccines	815 (63.3)	
In favor of vaccines	1007 (78.2)	
Do you follow social networks that generate doubts about vaccines?, n (%)	94 (7.28)	1291
Do you follow any social media profile? n (%)		
Against vaccines	99 (7.67)	1291
In favor of vaccines?	479 (37.1)	1290
Have you made any comments on social networks? n (%)		
Against vaccines	31 (2.40)	1289
In favor of vaccines	351 (27.4)	1281

Among the respondents, 66% (850/1287) reported having encountered antivaccine information on social network; of those who received this information, 24.3% (205/845) had doubts about the administration of recommended vaccines, and among those who had doubts, 13.3% (27/203) rejected a vaccine recommended by a health care professional. Among those who rejected vaccines for children, 10 (58.8%) were women, 13 (59.1%) were aged between 40 and 59 years, and 17 (68%) had university degrees; vaccines were rejected for children with an average age of 9.1 years.

A total of 63.3% (815/1287) of the respondents reported having received opinions or comments against vaccines on social networks, and 78.2% (1007/1287) reported having received opinions or comments in favor. In addition, 7.3% (94/1291) reported following social network accounts that generated doubts about vaccines, and 7.7% (99/1291) reported following antivaccine profiles on social network while 37.1% (479/1290) followed provaccine profiles on social network. A total of 2.4% (31/1289) stated they had made a comment against vaccines on social network, and 27.4% (351/1281) reported having made a comment in favor.

### Social Network Results

#### Sentiment Analysis

The analysis revealed the following distribution: 17.37% (83,357/479,734) of posts were classified as “Positive.” A significant portion, 54.44% (261,183/479,734) of posts, were categorized as “Negative.” Neutral sentiments accounted for 28.18% (135,194/479,734). When comparing with a human coder for positive and negative posts on a sample of posts, percent agreement was 75.24%, indicating the classifier had decent surface-level reliability. These findings suggested that the content in posts was likely to be more negative in nature (Table 3). Furthermore, the overall average sentiment was negative, with a result of −1.96 (SD 4.39).

**Table 3.** Classification of sentiment (N=479,734).

Sentiment	Value, n (%)
Positive	83,357 (17.37)
Negative	261,183 (54.44)
Neutral	135,194 (28.18)
Total	479,734 (100)

#### Location-Based Analysis

**Figure 1.**
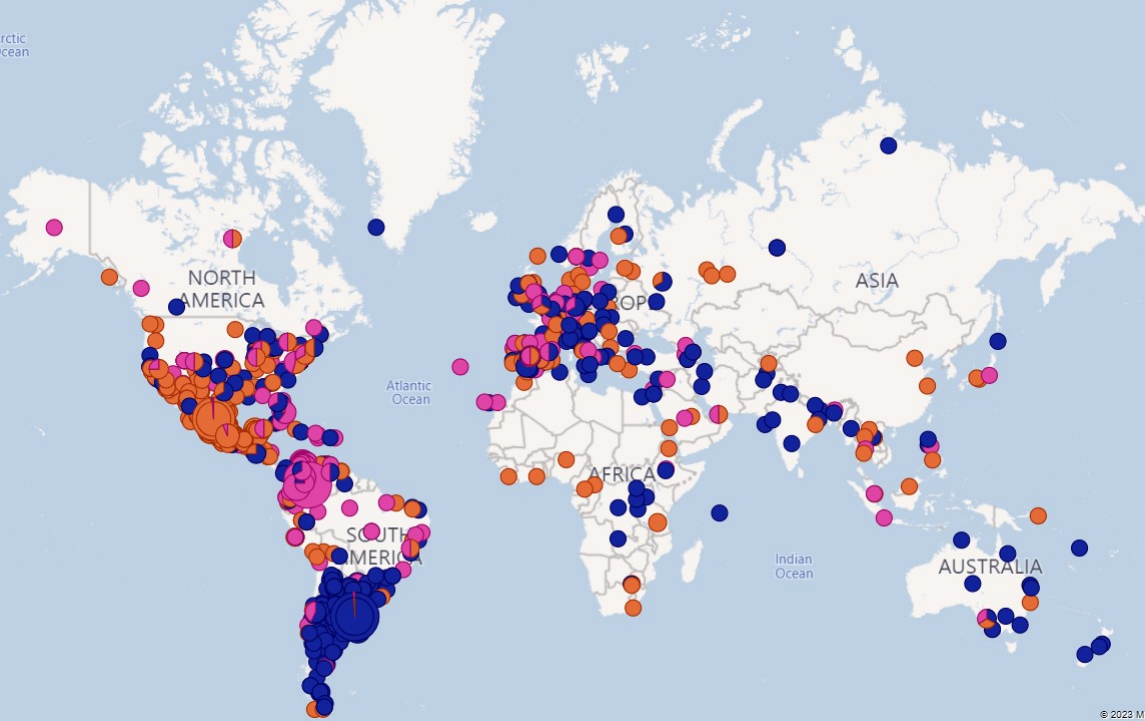
Overview of positive (orange), negative (dark blue), and neutral (purple) posts.

Location-based analysis focused on examining a specific time period in our dataset, that is, April 2021, as shown in Figures1  2; Most posts were from South America (such as Argentina, Venezuela, Colombia, and Mexico) and Europe (mainly Spain). Some posts were from other European countries, such as the United Kingdom and Germany.

**Figure 2.**
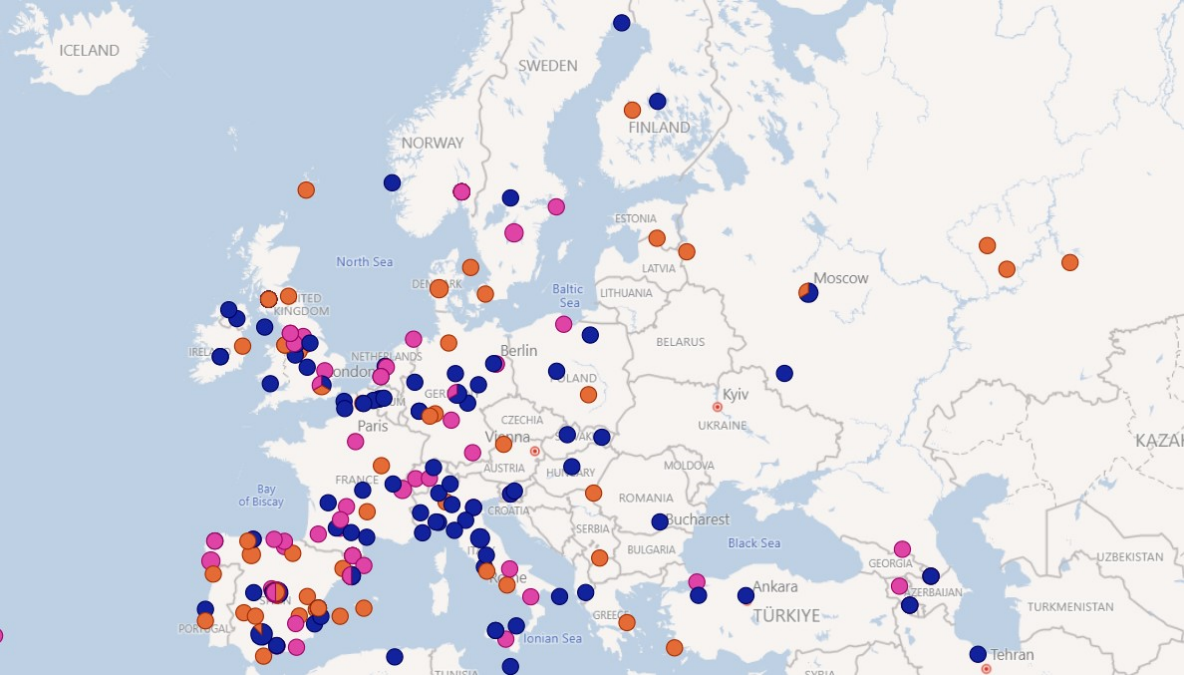
Zooming into Europe (mixed sentiment).

We observed a significant concentration of users in regions where Spanish was predominantly spoken. As indicated, these posts originated from South American countries, such as Argentina, Venezuela, Colombia, and Mexico. This trend underscored the Spanish-speaking demographic within these nations. In addition, a notable volume of posts was traced back to Europe, particularly Spain, further demonstrating the Spanish language’s influence when retrieving the data, as Spanish keywords were used to retrieve posts.

To gain an insight into the distribution of positive, neutral, and negative posts, further analysis was conducted using data captured during April 2021. The positive, neutral, and negative groups were identified during the social network analysis. Figures1  2 (April 2021) provide a visual overview of the locations. Sentiment analysis revealed regional differences across the geographic distribution of posts. More negative sentiments were predominantly observed in South America, while sentiment in Europe showed a mix of positive, neutral, and negative posts. In contrast, posts from North America exhibited comparatively more positive sentiments. Although the dataset included only Spanish- and Catalan-language posts, the presence of North America in the maps reflects the global reach of these languages, encompassing regions such as Mexico and Hispanic communities in the United States. It is important to highlight that location data were voluntarily self-reported by users, and thus geographic coverage may be incomplete or biased. These findings illustrate the varied landscape of vaccine-related discourse across Spanish- and Catalan-speaking populations during the study period.

#### Social Network Analysis and Topic Insight

The most prominent group identified was the “isolates” group (Group 1). This group was characterized by individuals who sent original posts without interactions. The predominance of this group suggested that the topics were widely popular and managed to draw a variety of unique and original opinions from a diverse audience.

**Figure 3.**
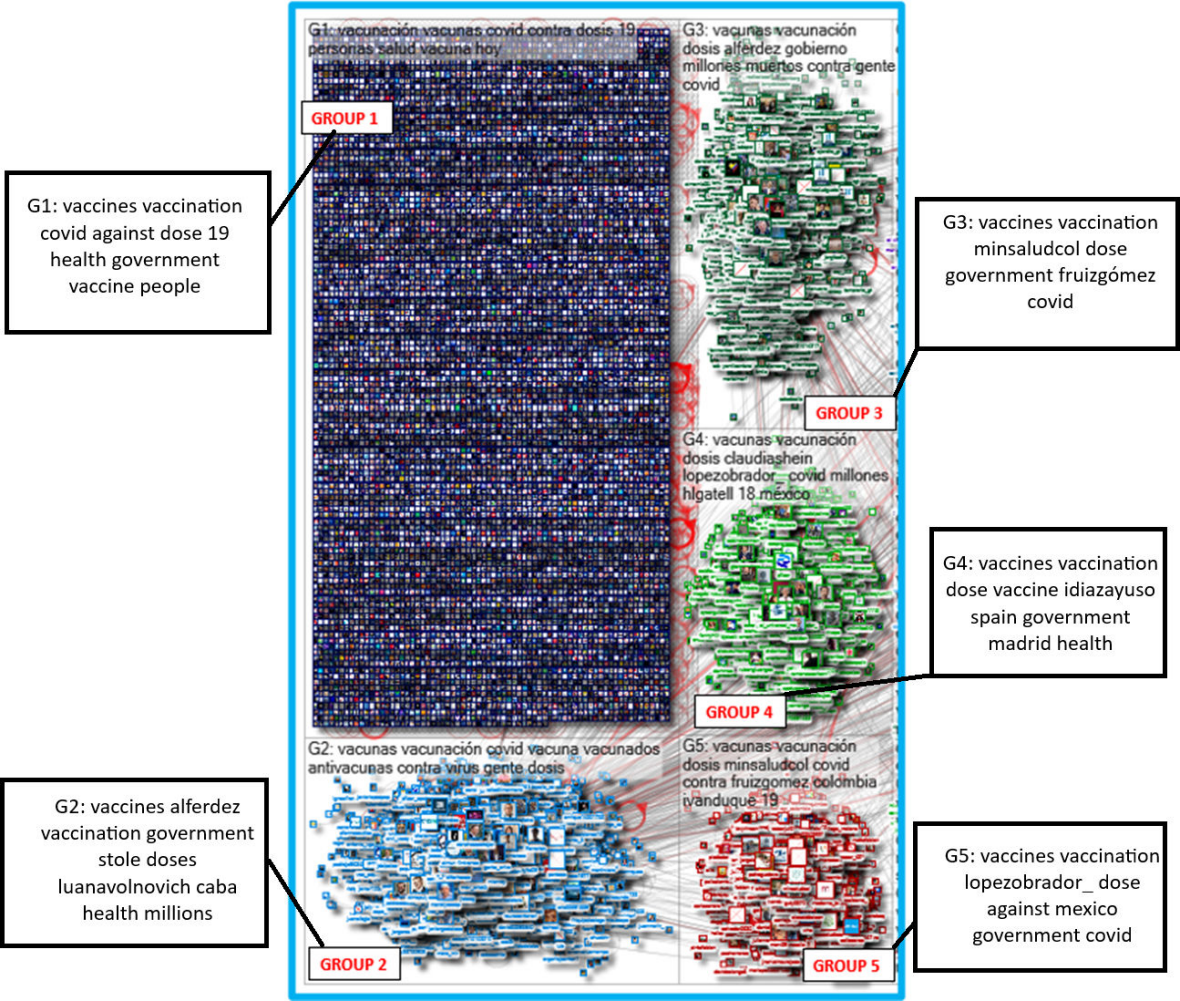
Social network analysis with top 5 groups (April 2021).

The groups numbered 3-5 exhibited characteristics of community network shapes intertwined with elements of broadcast. These groups demonstrated high interconnectedness, indicative of frequent and active back-and-forth conversations. At the same time, there was a noticeable amplification of certain accounts within these groups, which pointed to a blend of personal interaction and wider broadcast of information or opinions (Figure 3).

Table 4 provides insight into the top 5 users ranked by betweenness centrality, a metric indicating the extent to which a user acts as a bridge within a network. Alberto Fernández, the former Argentine President, had a very high centrality score. This suggested that he was a central user within the network and was key to information flow despite having fewer followers than global organizations like the World Health Organization (WHO). Furthermore, an ordinary citizen, labeled as a “Member of the Public,” ranked third, which highlighted how ordinary users can become influential on social media. User 4, the Pan American Health Organization, held a strong intermediary role despite a modest follower base. User 5, Iván Duque, had a notable influence through his centrality.

**Table 4.** Top users ranked by betweenness centrality.

Rank	User	Betweenness centrality	Followers
1	Alberto Fernández (Former President of the Argentine Republic [2019-2023])	127,116,284.117	2,304,550
2	The World Health Organization	48,398,978.460	12,356,193
3	Member of the Public	47,989,441.412	1715
4	The Pan American Health Organization (regional office for the Americas of the World Health Organization	47,361,720.241	430,494
5	Iván Duque (President of Colombia from 2018 to 2022)	39,020,770.895	2,565,413

The following conversation groups on X were analyzed:

1. Group 1: By examining the keywords and network insights, conversations centered around the global response to the COVID-19 pandemic, and they emphasized vaccination efforts. Users discussed the vaccination campaign targeting various groups, particularly older adults, who were among the most vulnerable to the virus.
2. Group 2: Discussions indicated challenges pointing to vaccine theft issues and highlighted the struggles in managing and securing the vaccine supply. There were also discussions on organized strategies and public health initiatives to combat the pandemic.
3. Group 3: Discussions were based on a focused campaign in the State of Mexico (Edomex), and users discussed the scale and nature of the initiative, which was the large-scale distribution of vaccines. There was also a personal, community-centric dimension to the campaign, which emphasized the societal importance of protecting older adults against COVID-19.
4. Group 4: There was a focused narrative on the COVID-19 pandemic, particularly highlighting the vaccination efforts for older adults. Users also referred to the “Chinese vaccines,” which implied a specific type of vaccine being discussed. Central themes included the process and challenges of vaccination, the procurement of vaccines, and the scale of the effort.
5. Group 5: The keywords suggested a comprehensive national plan in Colombia to combat COVID-19 through a vaccination campaign. The discussions also noted the well-defined procedure for administering the vaccines. There were also discussions of a targeted approach toward vaccinating older adults, particularly those more than 80 years old, recognizing their vulnerability to the virus. There were also discussions around the involvement of the Colombian Ministry of Health and its officials, including Fernando Ruiz Gómez, in orchestrating and overseeing the public health initiative (Table 5).

**Table 5.** Overview of topic keywords (April 2021).

Group	Key themes inferred
Group 1	Vaccination campaign and logistics Targeted vaccination for older adults Scale of vaccination effort
Group 2	Vaccine mismanagement and theft Vaccination of priority groups and health care workers Very Important Person vaccination controversy
Group 3	Alfredo Del Mazo’s administration’s role Nationwide vaccination against COVID-19 Community focus in vaccination
Group 4	Chinese vaccines and procurement Vaccination process and challenges Health care capacity and response
Group 5	Comprehensive National Vaccination Plan Special attention to vulnerable populations Ministry of Health’s involvement

In regard to the sentiment across the groups, it was not possible to quantify an entire group as either positive, negative, or neutral due to the diverse range of conversations taking place.

## Discussion

### Principal Findings

This study used a dual-methods design by combining a population-based survey in Spain with sentiment analysis of Spanish-language posts on X. Although these 2 components focused on different populations, survey respondents in Spain and global Spanish-speaking social media users, they provided complementary insights. The survey allowed us to identify who engaged with vaccine information on social networks and their characteristics, while the analysis of X offered a broader perspective on the type of content to which these users may be exposed. Given the transnational nature of social media, vaccine-related narratives circulate beyond national borders, influencing public perceptions regardless of their geographical origin. This exploratory approach facilitates a more comprehensive understanding of the potential impact of social networks on vaccine information access and perception. It is well-known that social media platforms are sources of health information and can have considerable influence on health decision-making [36]. In our study, we observed that a significant portion of the surveyed population in Spain reported frequent engagement with vaccine-related content on social media platforms, and 66% (850/1287) of respondents reported encountering antivaccine information. Concurrently, our analysis of Spanish- and Catalan-language posts on X identified that 54.44% (261,183/479,734) of posts were categorized as expressing negative sentiment. It is important to note, however, that negative sentiment does not necessarily indicate antivaccine attitudes. Such messages may include expressions of concern, criticism of public health measures, or institutional communications reporting unfavorable epidemiological data [37,38]. Previous research has shown that negative sentiment in vaccine-related posts can reflect a wide range of perspectives, including anxiety about side effects, mistrust in institutions, or reactions to the broader pandemic context. These findings highlight the importance of interpreting sentiment classifications with caution, as negative sentiment may encompass diverse forms of content that are not inherently opposed to vaccination. A large-scale analysis of 9,352,509 English-language posts from 2020 identified that only 2.49% were explicitly antivaccination. However, the same study reported an increase in antivaccine sentiment on social media following the initial rollout of COVID-19 vaccines [21]. Similarly, another study analyzing English tweets from the United Kingdom and the United States between March and November 2020 reported negative sentiment in 27.95% and 30.57% of posts, respectively [24]. These data reinforce the need for nuanced interpretation of social media discourse, as platforms like X are both a source of verified information and a space where conspiracy theories and misinformation can also circulate [33,39].

In our analysis of social networks, we identified relational patterns within the studied groups on the topic of vaccination. Specifically, groups numbered 3-5 exhibited characteristics of community network shapes intertwined with elements of broadcast. These groups demonstrated high interconnectedness, indicative of frequent and active back-and-forth conversations, mixing positive, negative, and neutral sentiments. A previous publication shows that interaction between antivaccine groups and undecided groups could facilitate vaccination hesitancy in the undecided due to exposure to antivaccine messages [15].

The analysis of each group allows us to see the key themes of the conversations, which can be useful for detecting the population’s concerns and creating vaccination campaigns aimed at combating misinformation or doubts about vaccination, potentially generating new evidence in community interventions as described in previous publications [13,16,22,24,40]. Some of these publications propose a method to counteract fake news, which involves quickly detecting and directly addressing it as it arises [33,39]. The analysis of topic words and key themes in our study allowed us to group the predominant themes of the 5 study groups, which were (1) vaccination efforts for the COVID-19 pandemic, (2) vaccine theft issues and struggles in managing and securing the vaccine supply, (3) a campaign in the State of Mexico, (4) vaccination efforts for older adults, and (5) a vaccination campaign in Colombia to combat COVID-19. We hypothesize that the topic words and themes may be influenced by the demographic distribution of posts from Spanish-speaking countries, primarily in South America. Another study also analyzed the themes of vaccine-related tweets on X in the Australian population, primarily detecting three themes, including attitudes and actions toward COVID-19 and its vaccination, infection control measures, and confidence in COVID-19 vaccine trials, alongside baseless claims, conspiracy theories, complaints, and misconceptions about various measures against COVID [23]. Another study develops algorithms for automatically classifying a large number of vaccine-related tweets into 3 classes, such as provaccine, antivaccine, and neutral [16].

The geographical location of the messages and the analysis of positive, negative, and neutral sentiments are useful tools for generating vaccination campaigns and detecting groups with higher hesitancy [24]. In our study, we found that more posts classified as negative appeared to derive from South America, with a mix in Europe, and more positive posts in North America. In Spain, positive and neutral posts predominated, which aligns with Spain generally showing good vaccination coverage and acceptance among the population [41]. However, given the high level of technological connectivity in today’s society, a tweet or post generated in North or South America could influence the Spanish population.

To combat vaccine hesitancy, it is essential to implement interventions that address the infodemic, such as promoting digital literacy and equipping the public with skills to critically evaluate digital media content [20,42]. Social media users often struggle to distinguish accurate information from misinformation, as they may be influenced by nonscientific influencers or fear-based narratives rather than validated scientific evidence [42]. Policy makers and public health care professionals must consider the adverse effects of social media on vaccine perceptions. Strategies to reduce vaccine hesitancy should include monitoring emerging trends, engaging with social media communities, and providing clear, evidence-based information [43]. Collaborating with trusted social media influencers to disseminate accurate health messages can also be an effective approach to counteract misinformation and build public trust [44].

A limitation of our study is that the “Social Network Analysis and Topic Insight” focused specifically on the X network in April 2021, so our findings may not be applicable to other time periods. Future research could extend the analyzed time periods and examine X discussions in different locations. One limitation of our sentiment analysis is the use of fixed sentiment categories (positive and negative). While this approach is widely used, it may not capture the full emotional spectrum of posts related to vaccination, such as feelings of sadness, anger, or fear. Future studies could expand the emotional categories used in sentiment analysis to provide a more comprehensive understanding of the diverse emotional responses to vaccine-related content on social media. A further limitation lies in the use of “self-reported” user bias, as users do not always specify their correct place of residence. In addition, certain accounts repeatedly tweeted and dominated discussions; future research could examine their role and impact within our dataset. Finally, we acknowledge that the selection of keywords may introduce bias in data collection. Future research should refine keyword selection strategies to improve representativeness across diverse Spanish-speaking regions and assess potential biases arising from linguistic variations.

One of the limitations of the survey was the inherent recruitment bias associated with the internet-based survey method, which restricted participation to individuals with internet access. However, given that 96.1% of Spanish households have internet access and 85% of Spaniards are users of social networks [45,46], the reach of our survey remains substantial. Another possible limitation is potential respondent repetition, although we anticipate minimal impact on the final results because of the expected low rate of repetition. In addition, our sample skewed toward women and people with a university education, which could limit the interpretation and generalization of our study to the broader population. Finally, reaching the antivaccine population posed challenges, as interactions with these groups are difficult. To mitigate eventual bias deriving from this, in our sample size calculation, we considered that the proportion of antivaccine responses would be much lower than the proportion of provaccine responses. Despite these limitations, our study provides valuable insights into opinions about vaccines, but caution is warranted in extrapolating findings to the entire population.

### Conclusions

Our study highlighted that a significant proportion of the surveyed population encountered antivaccine content on social networks. There was a notable presence of posts categorized as negative on the social network X, particularly from South America. These results underscore the need for nuanced interpretation of social media sentiment, as negative posts may reflect diverse concerns, including public health measures, vaccine safety, or broader pandemic-related issues. The integration of sentiment, thematic, and geolocation analyses may offer useful insights into the evolving nature of vaccine-related discourse on social media. While exploratory in nature, this approach can support public health efforts to monitor social media narratives and adapt communication strategies accordingly. Although further research is needed, our findings suggest the potential value of actions, such as improving digital health literacy, supporting critical appraisal of social media content, and providing timely, evidence-based information in response to misinformation trends. Public health institutions may also benefit from more direct engagement with social media communities to build trust and promote informed dialogue. These findings provide actionable insights that can inform ongoing digital surveillance, support the design of targeted public health messaging, and enhance risk communication strategies tailored to specific populations and critical moments. Recognizing the evolving nature of social media platforms, including Twitter’s recent rebranding to X, adapting these approaches will be essential for maintaining effective vaccine-related communication in dynamic social media environments.

## Supplementary material

10.2196/63223 Multimedia Appendix 1 Survey.

## Abbreviations

AI: artificial intelligence
REDCap: Research Electronic Data Capture
