# Supplementary material for: Mapping Vaccine Sentiment by Analyzing Spanish-Language Social Media Posts and Survey-Based Public Opinion: Dual Methods Study
Source: JMIR Infodemiology. 2025 Aug 29;5:e63223. doi: 10.2196/63223 (PMC12400125; doi:10.2196/63223)
Supplement: Multimedia Appendix 1 [file infodemiology-v5-e63223-s001.pdf]

SOCIOCULTURAL FACTORS

1. Your religious beliefs are:

□ Opposed to vaccination

□ In favour of vaccination

□ I am not a believer

□ I don’t know

2. Your culture is:

□ In favour of vaccination

□ Opposed to vaccination

□ Does not express a clear stance

□ I don’t know

3. Your family is:

□ In favour of vaccination

□ Opposed to vaccination

□ Does not express a clear stance

□ I don’t know

4. What is your highest level of education?

□ No formal education or incomplete primary education

□ Primary education

□ Secondary education

□ Baccalaureate

□ Vocational training / Professional qualification

□ University education

5. You are:

□ Man

□ Woman

□ Other

6. Do you have children aged 14 or under?

□ Yes

□ No

7. What is your date of birth?

____ / ____ / _______

8. What is the postcode of your current residence?

INFORMATION ON SOCIAL MEDIA USE

1. Are you a regular social media user?

□ Yes

2. Since when?

□ For less than 1 year

Since the COVID-19 lockdown? □ Yes □ No

□ For 1 to 5 years

□ For more than 5 years

3. Have you searched for information about vaccines on social media?

□ Yes

4. On which social media platforms?

Twitter □ Yes □ No

Instagram □ Yes □ No

Facebook □ Yes □ No

Others: ___________

5. In which year did you first search for vaccine-related information on social media?

6. In which year did you last search for vaccine-related information on social media?

7. Was this search related to COVID-19?

□ Yes

□ No

8. Have you come across information AGAINST vaccines on social media?

□ Yes

9. Did this information make you question whether to accept recommended vaccinations?

□ Yes

10. Has information found on social media led you to reject any vaccine recommended by a healthcare professional?

□ Yes

11. If you rejected a vaccine for a child, what was the child's age?

12. If a vaccine was rejected for a child, who initially had doubts?

□ Yourself

□ Someone else

If someone else, please specify: ___________

13. Gender of the person who rejected the vaccination:

□ Woman

□ Man

□ Other

14. Age of that person who rejected the vaccination:

□ Under 30

□ 30–39

□ 40–49

□ 50–59

□ 60–69

□ Over 70

15. Highest level of education completed by the person who rejected the vaccination:

□ No formal education or incomplete primary education

□ Primary education

□ Secondary education

□ Baccalaureate

□ Vocational training / Professional qualification

□ University education

18. Have you received any comments or opinions AGAINST vaccines via social media?

□ Yes

□ No

19. Have you received any comments or opinions IN FAVOUR of vaccines via social media?

□ Yes

□ No

20. Do you follow any social media accounts that make you question vaccines?

□ Yes

□ No

21. Do you follow any social media accounts that are against vaccines?

□ Yes

□ No

22. Do you follow any social media accounts that are in favour of vaccines?

□ Yes

□ No

23. Have you ever posted a comment on social media AGAINST vaccines?

□ Yes

□ No

24. Have you ever posted a comment on social media IN FAVOUR of vaccines?

□ Yes

□ No
